# The Protective Performance of Modern Motorcycle Helmets Under Oblique Impacts

**DOI:** 10.1007/s10439-022-02963-8

**Published:** 2022-04-13

**Authors:** Xiancheng Yu, Ingrid Logan, Itziar de Pedro Sarasola, Atulit Dasaratha, Mazdak Ghajari

**Affiliations:** 1 Dyson School of Design Engineering, Imperial College London, South Kensington, London, SW7 2AZ UK; 2 Department of Mechanical Engineering, Imperial College London, South Kensington, London, SW7 2AZ UK

**Keywords:** Motorcycle helmet, Oblique impact, Brain injury, Head injury, Rotational acceleration

## Abstract

Motorcyclists are at high risk of head injuries, including skull fractures, focal brain injuries, intracranial bleeding and diffuse brain injuries. New helmet technologies have been developed to mitigate head injuries in motorcycle collisions, but there is limited information on their performance under commonly occurring oblique impacts. We used an oblique impact method to assess the performance of seven modern motorcycle helmets at five impact locations. Four helmets were fitted with rotational management technologies: a low friction layer (MIPS), three-layer liner system (Flex) and dampers-connected liner system (ODS). Helmets were dropped onto a 45° anvil at 8 m/s at five locations. We determined peak translational and rotational accelerations (PTA and PRA), peak rotational velocity (PRV) and brain injury criteria (BrIC). In addition, we used a human head finite element model to predict strain distribution across the brain and in corpus callosum and sulci. We found that the impact location affected the injury metrics and brain strain, but this effect was not consistent. The rear impact produced lowest PTAs but highest PRAs. This impact produced highest strain in corpus callosum. The front impact produced the highest PRV and BrIC. The side impact produced the lowest PRV, BrIC and strain across the brain, sulci and corpus callosum. Among helmet technologies, MIPS reduced all injury metrics and brain strain compared with conventional helmets. Flex however was effective in reducing PRA only and ODS was not effective in reducing any injury metrics in comparison with conventional helmets. This study shows the importance of using different impact locations and injury metrics when assessing head protection effects of helmets. It also provides new data on the performance of modern motorcycle helmets. These results can help with improving helmet design and standard and rating test methods.

**Supplementary Information:**

The online version contains supplementary material available at 10.1007/s10439-022-02963-8.

## Introduction

Motorcyclists are at high risk of injuries in road traffic collisions. The 2019 annual report of road casualties in Great Britain shows that the fatality rate per passenger mile for motorcyclists is 65 times larger than car occupants.^49^ The same report highlights that the number of motorcyclist fatalities have fluctuated between 319 and 365 over 2011 to 2019, without indicating a clear trend. According to the US government in 2019, 5014 motorcyclists died on the roads, accounting for 14% of all vehicle crash deaths of the year while motorcycles form only 3% of all registered vehicles.^2^ Traumatic brain injury (TBI) is the leading cause of death and disability in motorcyclists.^42^ In motorcycle accidents, the head can impact another vehicle, infrastructure or road surface at high speeds, leading to skull fractures, intracranial bleeding and diffuse axonal injuries (DAI). A recent analysis of Great Britain’s Road Accident In-Depth Studies (RAIDS) database showed that of the 267 motorcyclists involved in collisions between 2013 and 2020, 8.3% sustained a skull fracture, 3.7% sustained a subdural haematoma, 10.1% sustained a subarachnoid haematoma and 9.4% sustained a focal brain injury.^6^ The analysis did not report the rate of DAI as the clinical data in RAIDS are based mainly on CT imaging, which often misses diffuse axonal injuries.^6,46^ The vast majority of the motorcyclists wore a helmet as mandated by law.

Helmets are the only equipment that can protect the head in motorcycle collisions. The main function of helmets is to absorb the energy of the impact and reduce the impact force and translational acceleration of the head. Reducing the contact force can greatly reduce the risk of skull fractures and associated intracranial bleeding.^39^ In addition, reducing the translational acceleration of the head during impacts can help with reducing focal brain injuries.^38,51^ Impact forces, however, can also produce head rotations, which are shown to produce diffuse injuries and subdural haematoma (SDH).^14,40^ However, mitigation of head rotation has not been an objective in helmet design until recently.

**Figure 1:**
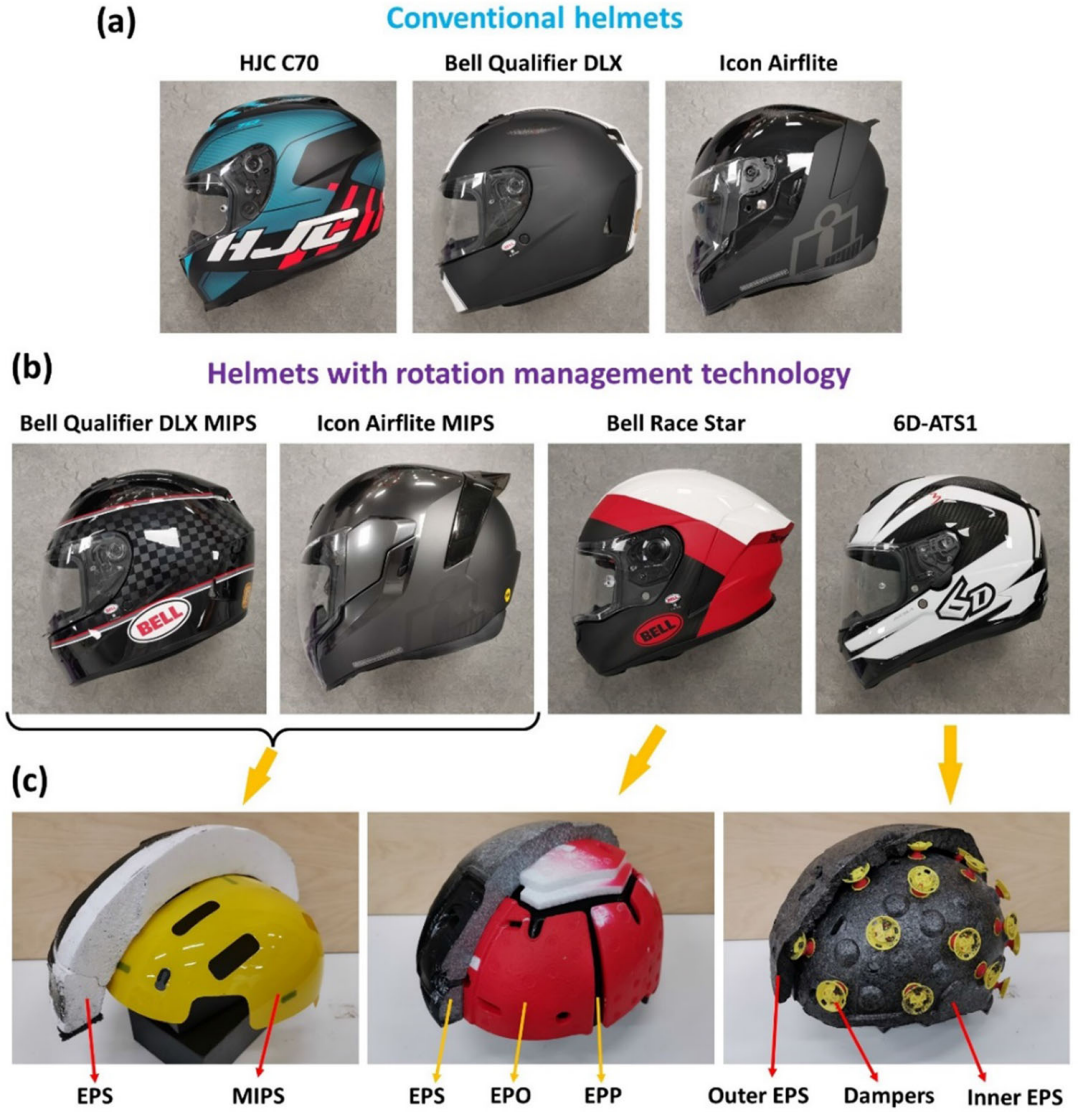
(a) Conventional helmets. (b) Helmets with rotation management technology. (c) Helmet technologies studied here: MIPS layer, Flex and ODS.

New helmet technologies are now available in the market, which are designed to manage the rotational motion of the head during head impacts.^1^ Figure 1 shows three popular technologies: Multi-Directional Impact Protection System (MIPS), Flex (three-layer impact liner) and Omni-Directional Suspension (ODS). MIPS is a slip-plane layer between the EPS (expanded poly styrene) liner and comfort liner. This low friction layer is designed to allow rotational movement between the head and helmet during impact, which potentially reduces the transfer of rotational energy to the head.^26^ The Flex impact liner is made of three layers with different energy absorbing materials: EPS for outer layer, EPO (Expanded Polyolefin) for the middle layer and EPP (expanded polypropylene) for the inner layer. According to the manufacturer, this progressive layering technology aims on reducing both translational and rotational impact energies.^50^ During oblique impacts, the middle layer of the liner is supposed to act as a slip zone between the outer and inner layer and reduce the rotational energy transferred from the helmet to the head. The ODS technology has two layers of EPS liners connected with dampers.^28^ In the 6D helmet (model: 6D-ATS1), there are 27 dampers connecting the outer and inner EPS liners. The manufacturer suggests that the elastic properties and shape of the dampers allow the inner liner to displace and shear within the outer liner during impacts and mitigate rotational movement of the head. Although the manufacturers have claimed that their technology can mitigate brain injury, the performance of motorcycle helmets fitted with these new technologies are yet to be assessed.

Accident data show that the majority of motorcycle head collisions are oblique, i.e. the impact speed has a component tangential to the impact surface.^17,44^ One study reconstructed 56 motorcycle accidents and reported that the average head impact angle, defined as the angle between the head impact velocity and impact surface, is 44°.^10^ A recent study investigated the effect of head impact angle on brain deformation.^45^ Their results showed that a head impact angle of 30° to 45° produces larger brain strains predicted by using an FE model of the human head than other angles. Two recently released motorcycle helmet test standards have adopted a 45° anvil for oblique impacts, where the rotational acceleration is measured.^13,18,21^ The ECE22.06 motorcycle standard oblique impact test method requires dropping the helmet fitted onto the EN960 headform onto a 45° anvil at 8 m/s.^18^ The ECE22.06 standard requires two helmet samples to be tested at five locations, rotating within the transverse plane. The Fédération Internationale de Motocyclisme (FIM) has also introduced an oblique impact test in their Racing Homologation Programme (FRHP) for motorcycle racing helmets.^21^ This oblique impact test is identical to the ECE22.06 method, except that the EN960 headform is coated with a layer of platinum cure silicone. This layer of coating increases the coefficient of friction between the helmet liner and headform from 0.16 to 0.78.^21,55^ Most recently, a French organization released the Certimoov helmet test rating for both bicycle and motorcycle helmets.^13^ Certimoov also uses an oblique impact test using a 45° anvil and 8m/s impact speed. It includes three impact locations, each producing dominant headform rotation about one of the anatomical axes of the head. In addition, it uses a Hybrid III headform. Despite the introduction of these oblique impact test methods, there is still limited information available regarding the performance of motorcycle helmets, particularly those fitted with new technologies, under oblique impacts. In addition, the effects of impact locations on injury metrics during oblique impacts on motorcycle helmets remain unknown.

In this paper, we studied the response of several modern motorcycle helmets under oblique impacts at five different locations. Our first aim is to determine the head and brain protection performance of the helmets using injury metrics based on translational and rotational motion of the head and brain tissue response based on finite element analysis. Our second aim is to determine whether the impact location affects helmet’s performance and whether this effect is consistent across injury metrics. Our third aim is to test if motorcycle helmets fitted with rotation management technologies can better protect the head and brain under oblique impacts compared with conventional helmets.

## Methods

### Motorcycle Helmets

We selected 7 commercial motorcycle helmets, available in the UK market. Their details are listed in Table 1. The size of the helmets was chosen to fit head circumferences of 57–58 cm. Three helmets do not include any specific technology for mitigating head rotation, which are categorised as conventional helmets and serve as baseline. The other four helmets have incorporated three rotational technologies: two helmets have MIPS; one helmet has the Flex impact layer and one helmet has ODS (Fig. 1). The two helmets with MIPS technology have their corresponding versions without MIPS. 6 samples of each helmet were purchased.

**Table 1:** Summary of the studied motorcycle helmets.

Helmets	Abbrev.	Shell material	Technology	Price^a^	SHARP rating
HJC C70	HJC^b^	Polycarbonate	–	£150	5-star
Bell Qualifier DLX	B.Q^b^	Polycarbonate	–	£180	3-star
Icon Airflite	I.A^b^	Polycarbonate	–	£230	3-star
Bell Qualifier DLX MIPS	B.Q-MIPS	Polycarbonate	MIPS	£210	3-star
Icon Airflite MIPS	I.A-MIPS	Polycarbonate	MIPS	£260	–
Bell Race Star	B.R.S-Flex	CFRP^c^	Flex	£400	5-star
6D-ATS1	6D-ODS	CFRP^c^	ODS	£550	–

^a^Prices are either taken from the SHARP website (for SHARP rated helmets) or the recommended retail price from the manufacturer’s website

^b^Conventional Helmets (no rotation management technology) serve as baselines

^c^*CFRP* carbon fibre reinforced polymer

Five helmets have shells made from polycarbonate, with prices less than £260. Helmets with MIPS are £30 more expensive than their non-MIPS version. The helmets fitted with Flex and ODS technologies have shells made from carbon fibre reinforced polymer (CFRP) composites, bringing the price to over £400. Five out of seven helmets have been rated by the Safety Helmet Assessment and Rating Programme (SHARP), the motorcycle helmet rating program introduced by the UK Department for Transport in 2007.

### Oblique Impact Tests

The oblique impact tests were conducted with the drop tower helmet test rig at the Human Experience, Analysis and Design (HEAD) lab, Imperial College London. The rig was designed to perform both linear and oblique impacts on different types of helmets, e.g. motorcycle, bicycle, ski *etc*. We followed the oblique impact test method recently introduced in the ECE22.06 motorcycle helmet testing standard to conduct the tests,^11^ except that we used a Hybrid III (HIII) 50th percentile male dummy headform. The mass and the circumference of the headform is 4.54 kg and 57.2 cm, respectively.^30,35^ This headform has been used in a number of previous studies as its mass and moment of inertia better represents the 50th percentile human head.^1,29,57^

**Figure 2:**
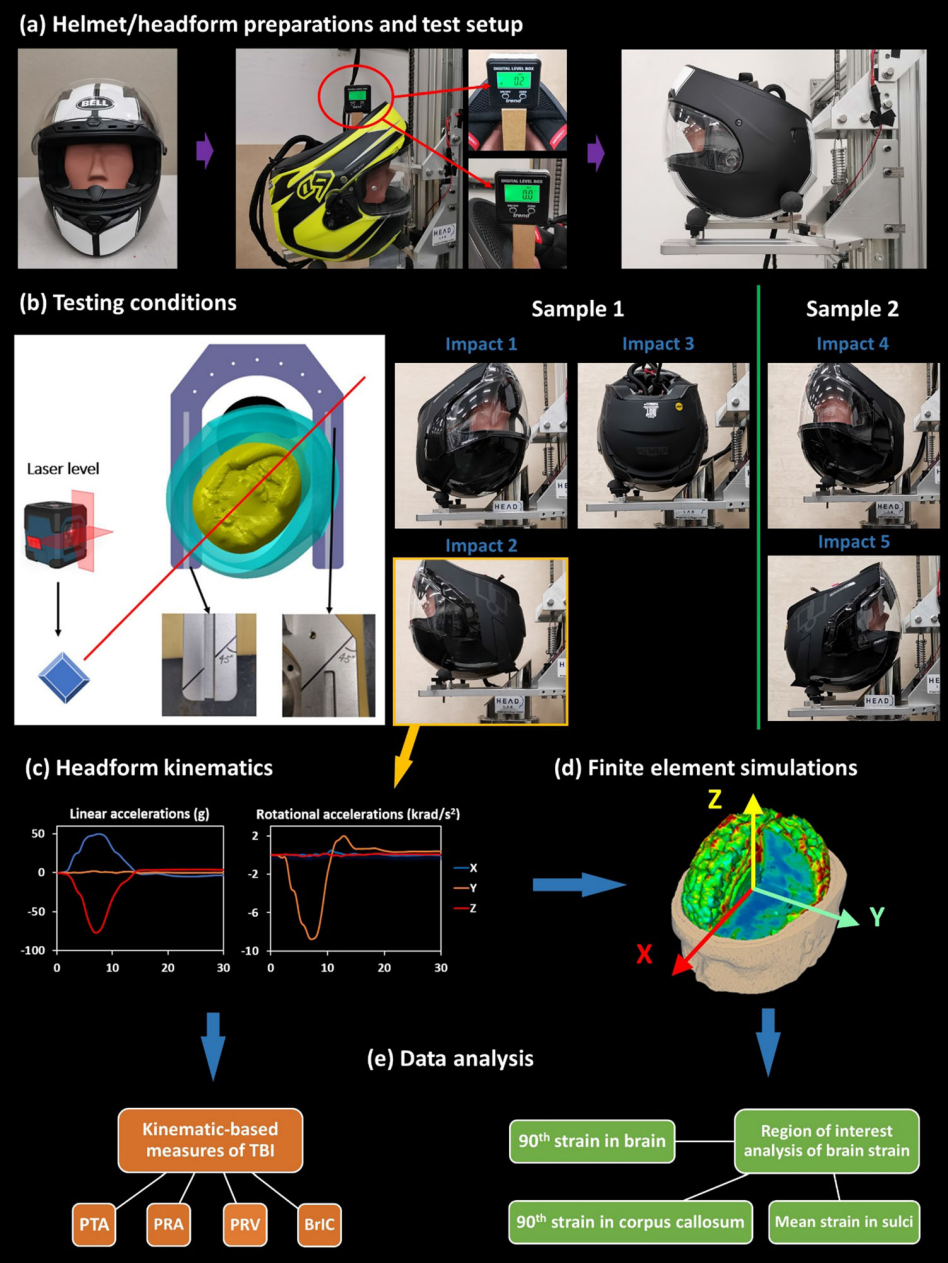
Experimental and computational methods. (a) Helmet/headform preparation and test setup. (b) A laser level was used for placing the helmet for different impact location. Each helmet was tested at five impact points. (c) For each test, three translational and three rotational acceleration time-history data were recorded with the HIII headform. (d) These acceleration data were then applied to a detailed finite element model of human head to determine the brain strains. (e) Finally, we performed data analysis on the kinematics-based injury metrics, calculated from the acceleration data, and the brain strain in the entire brain and key regions: sulci and corpus callosum.

The helmet was fitted onto the HIII headform based on the helmet fitting requirements of ECE22.06. The helmeted headform was then placed on a free-falling U-shape testing platform (3.2 kg). Using an inclinometer, we adjusted the headform orientation so that the bottom of the headform was nearly horizontal (< 0.5°) (Fig. 2a). Then, we used masking tape to fix the helmet onto the platform, which maintained the helmet’s position and orientation during the free fall. The masking tape was pre-cut at several points to ensure an easy tear during the impact. We have verified that the tape fixture has minimal affect in the headform motion (supplementary material Appendix 2).

The helmeted headform was dropped onto a 45° anvil at a speed of 8.0 (+ 0.15/− 0.0) m/s.^11^ This speed has been adopted in the oblique impact test methods in ECE22.06 and FRHP. The 45° oblique anvil was manufactured from a 130 mm-diameter solid steel cylinder. The surface of the oblique anvil was covered with an 80-grit abrasive paper, which was replaced after each test. A high-speed video camera was placed behind the anvil to record the impacts at 1770 frames per second (fps) (Figure 2a). After each test, the high-speed video was checked to make sure the helmet maintained its orientation and position on the platform until it impacts on the oblique anvil. The acceleration data of each test was also checked and compared to the other repeats to check the repeatability of the tests. Particularly, we checked that the helmet was not displaced on the platform during the free fall and before the impact.

Each helmet was tested at five impact locations using 2 samples, as shown in Figure 2b. The angle between the headform’s sagittal plane and the anvil middle plane ranged from 45° to 180°. Impact 1 to 3 were performed on helmet sample 1, and Impacts 4 and 5 were performed on helmet sample 2. The closest impact points (i.e., impacts 2 and 3) are at least 90° apart from each other. This is to make sure that the impact locations were separated from each other to minimize the influence of accumulated damage on the subsequent tests. Each test was repeated three times with three samples. Therefore, each helmet required 15 tests on 6 samples.

A nine-accelerometer package (NAP) was installed inside the headform to measure translational and rotational accelerations of the headform (Figure 2c). The accelerometers were arranged in a 3-2-2-2 array.^52^ The accelerations were sampled by a datalogger at 50 kHz frequency. The data was then filtered using a fourth-order Butterworth filter at a cut-off frequency of 1 kHz.^23^ By integrating the rotational accelerations vs. time, we obtained the rotational velocities.

### Calculation of Kinematics-Based Injury Metrics

The kinematics data of the tests were processed to obtain four brain injury metrics: peak translational acceleration (PTA), peak rotational acceleration (PRA), peak rotational velocity (PRV) and brain injury criteria (BrIC).^54^ BrIC was calculated using the following equation:

1 $$BrIC=\sqrt{{(\frac{{\omega }_{x-max}}{{\omega }_{xC}})}^{2}+{(\frac{{\omega }_{y-max}}{{\omega }_{yC}})}^{2}+{(\frac{{\omega }_{z-max}}{{\omega }_{zC}})}^{2}}$$

where ${\omega }_{x-\rm{max}}$, ${\omega }_{y-\rm{max}}$ and ${\omega }_{z-\rm{max}}$ are the maximum rotational velocity about *X*, *Y*, and *Z* axes respectively. ${\omega }_{xC}$, ${\omega }_{yC}$ and ${\omega }_{zC}$ are the critical angular velocities in their respective directions, with values of 66.25, 56.45 and 42.87 rad/s respectively.^54^ PTA is used in all helmet standards and previous work shows that it can predict the risk of skull fractures and focal brain injuries.^3,25^ PRA has been suggested as a metric for predicting SDH.^14,22^ PRV and BrIC have been shown to predict the risk of diffuse axonal injuries.^40,54^ Since these pathologies are seen in motorcycle casualties,^6^ we used all four metrics to evaluate the performance of the helmets.

### Finite Element Prediction of Strain Distribution

To predict patterns of mechanical strain across the brain, we used the Imperial College finite element (FE) model of the human head, developed in our previous study^24^ (Figure 2d). The model includes 11 tissues with detailed definition of their anatomy, e.g., sulci. More details on the model, validation of its brain tissue displacement predictions against recent well-documented cadaver experiments and some of its applications can be found in our previous studies.^1,20,24,58–60^

We assumed that the human skull is rigid due to its small deformation in helmeted impacts. The three translational and three rotational accelerations measured in helmet experiments were applied to the skull at the centre of gravity of the head. The first 30ms after initial contact was simulated, which was enough for the brain to experience the peak strain. The simulations were conducted using the nonlinear hydro-code LS-DYNA R11.^27^

We recorded the nodal displacement and velocity vectors during the simulations at every 0.1ms. These data were then processed to determine the maximum value of the first principal Green-Lagrange strain of each element of the brain over the entire simulation time. The outputs were written in MRI (Magnetic Resonance Imaging) NIfTI (Neuroimaging Informatics Technology Initiative) format. The data was analysed with FSL, which is a comprehensive tool for MRI brain imaging data analysis.^32^ We determined the 90th percentile strain of the entire brain, which represents the overall brain response to the impact. We also determined the 90th percentile strain in the corpus callosum (CC), a major white matter tract where injuries are often seen,^37,53^ and the mean strain in the sulci, which is the location of the chronic traumatic encephalopathy (CTE) pathology.^4,24,41^ To determine the strain in CC and sulci, we first used Freesurfer (an open-source package for the analysis of neuroimaging data) to segment the structural MRI that we used to generate the head FE model. This process resulted in an accurate spatial map of the CC and grey/white matter boundary. The grey/white matter spatial map was further divided into 60 gyri and 66 sulci regions based on the Destrieux Atlas,^15^ which allowed us to calculate the strain within the anatomically correct sulcal regions. For each helmet at each impact location, we simulated the three repeated tests. Then, we calculated the strain data for each of the three repeats.

### Determining the Effect of Impact Location and Helmet Technology

First, we performed two-way ANOVA using the helmet type and impact location as the factors and the kinematics-based injury metrics and brain strains as the outcome measure.^1^ This allowed us to investigate the effects of helmet type and impact location on helmets’ protective performance, which is the second aim of this study.

Next, we investigated if new motorcycle helmet technologies can better protect the head, which is the third aim of this study. We placed the HJC, B.Q and I.A helmets, which have no rotation management technology, in one group referred to as conventional helmets. The B.Q-MIPS and I.A-MIPS were placed in one group for MIPS technology. B.R.S-Flex and 6D-ODS were the other two groups that used Flex and ODS technologies respectively. We conducted one-way ANOVA with technology as the factor to determine its effect on the kinematics-based injury metrics and brain strains. We also conducted pairwise comparisons (post-hoc) to evaluate the differences between the three technologies and group of conventional helmets in terms of each injury metric and brain strains, compared to the group of conventional helmets.

## Results

### Head Kinematics in Oblique Impacts

**Figure 3:**
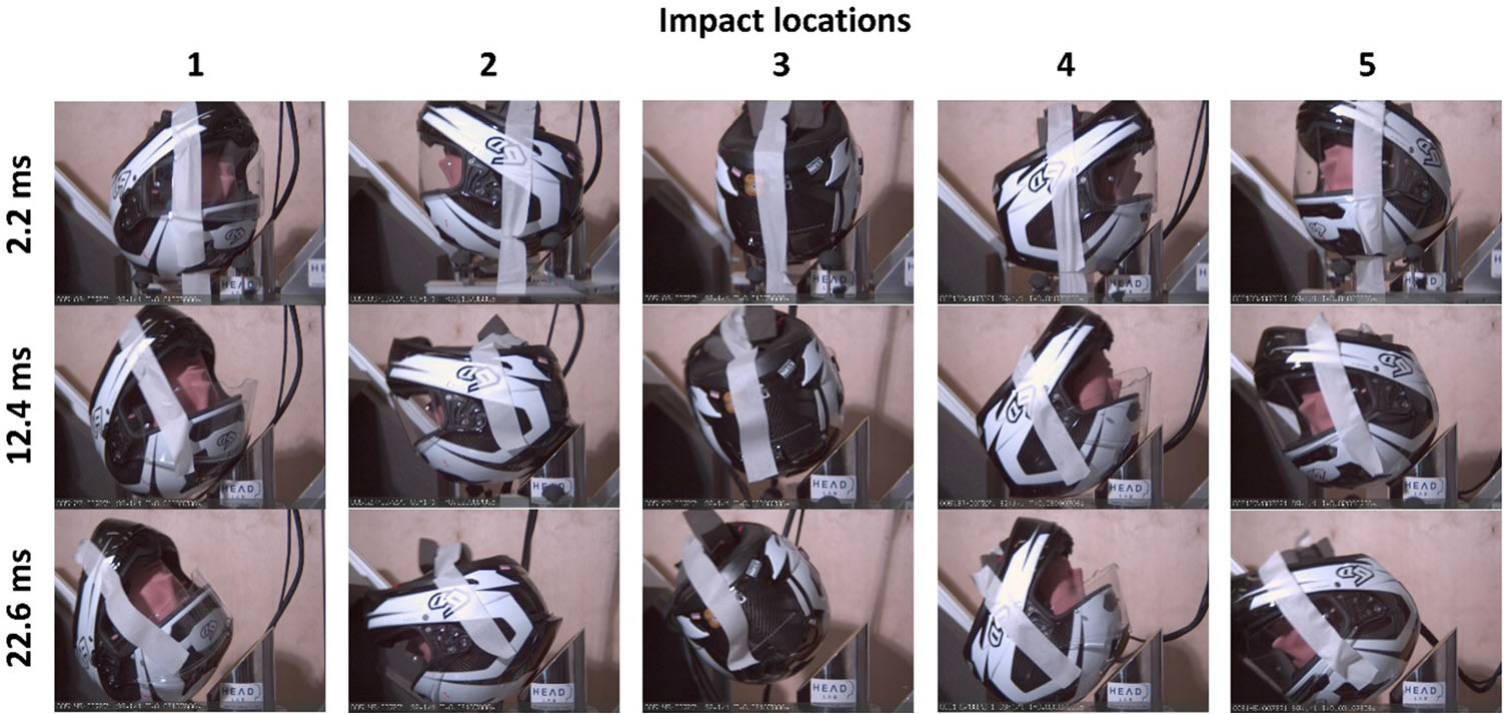
Snapshots from the high-speed videos of the 6D-ODS helmet under oblique impact at five different locations.

Figure 3 shows snapshots from high-speed videos of a helmet (6D-ODS) in five impact locations. As shown, the helmet rolled on the anvil shortly after the contact and then separated from the anvil. The helmet started to separate from the anvil around 12 ms after the contact initiation (Figure 3).

**Figure 4:**
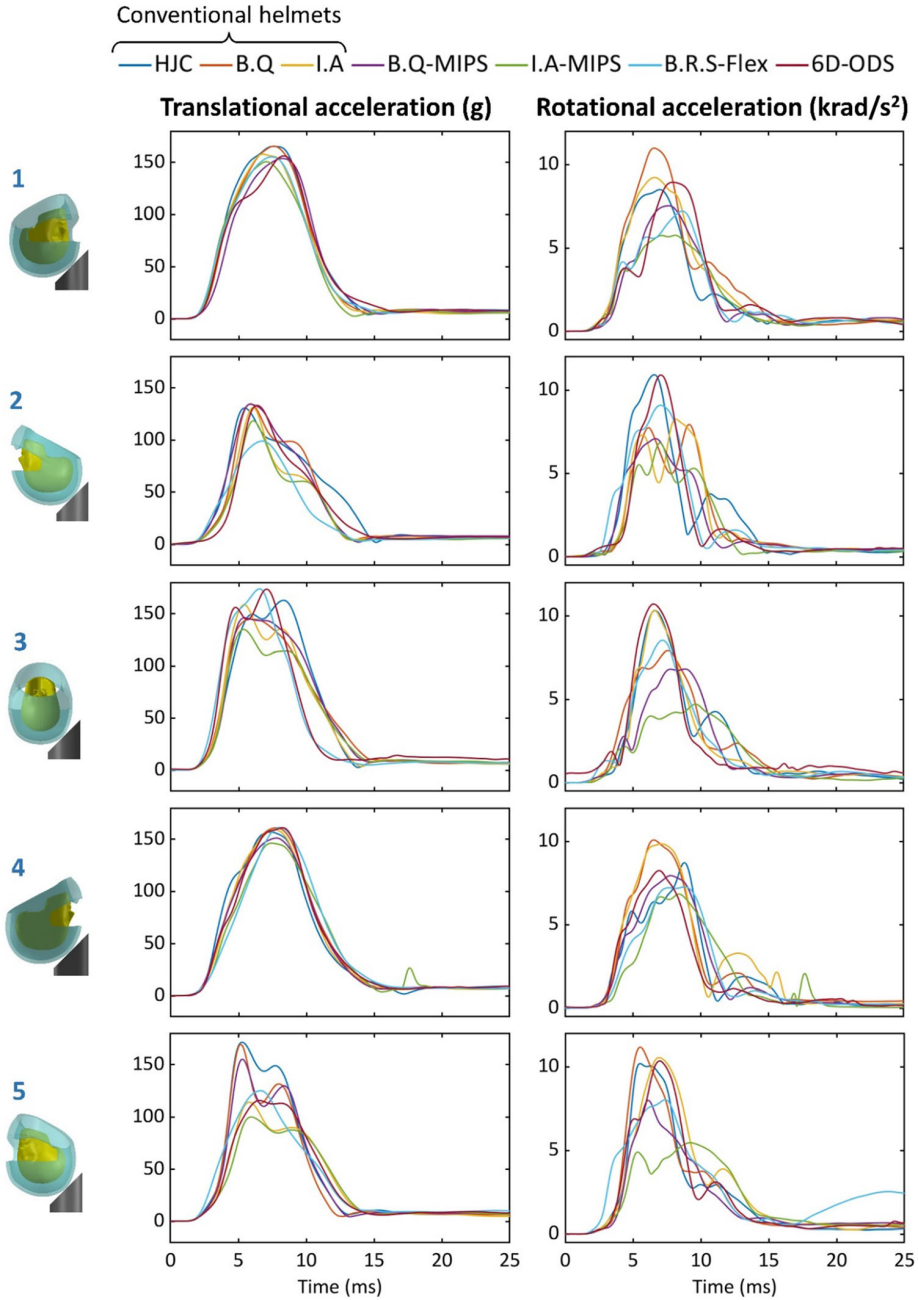
Mean resultant translational and rotational time-history. The results show that acceleration pulses peaked between 8 and 10 ms (after initial contact) with an impact duration under 15 ms.

Figure 4 shows the mean resultant translational and rotational acceleration time histories for all helmets under all impact conditions. These acceleration histories were averaged from the 3 repeats. The translational and rotational accelerations vary significantly between helmets and across impact locations. Noticeably, impact 2 (rear impact) produced lower translational accelerations compared with other impact locations. The impact duration for all impact locations and helmets was between 12 and 15 ms.

**Figure 5:**
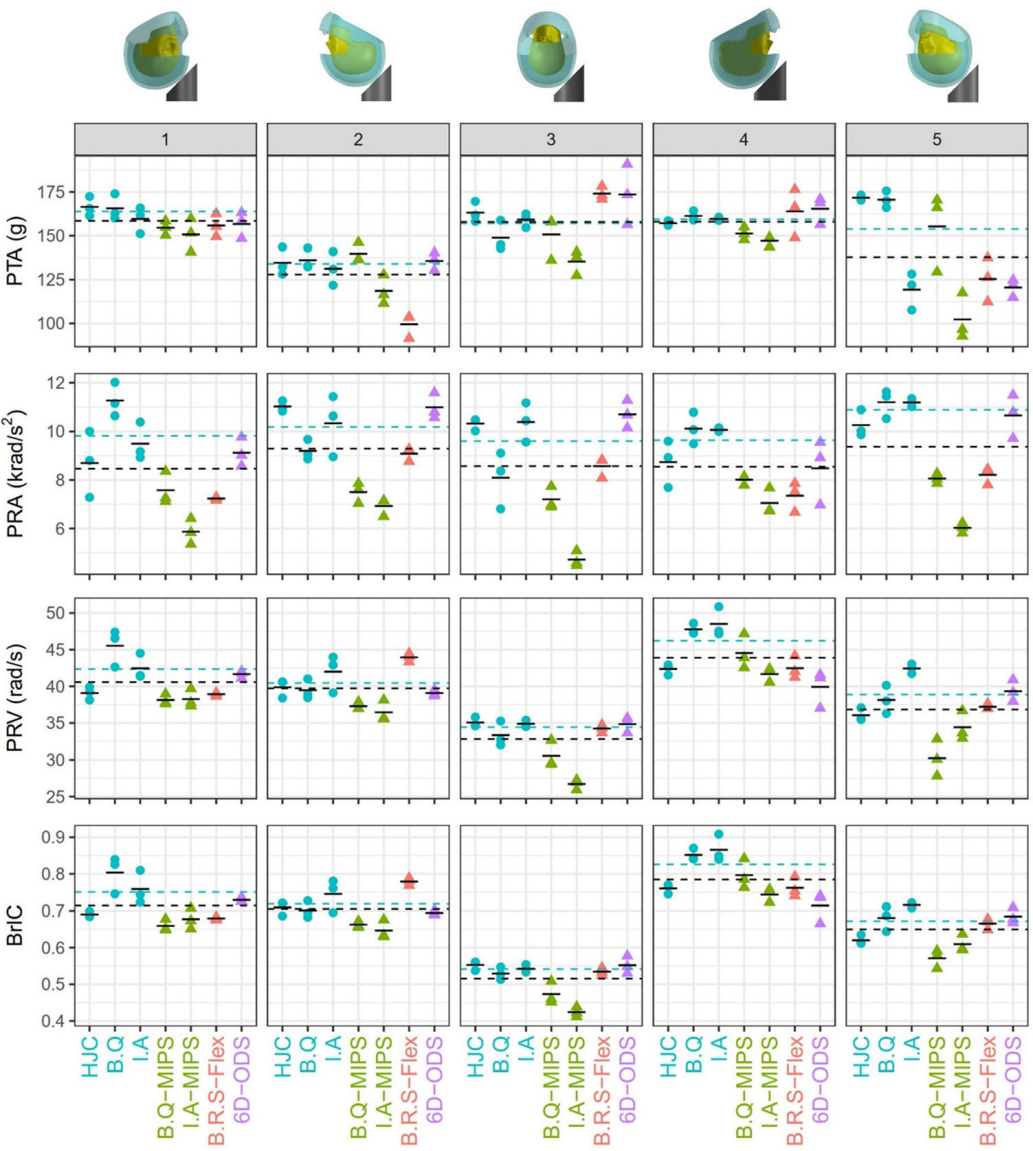
Injury metrics for all helmets at all impact locations. The plot shows three repeats (markers) and the mean value (broken black line) of each helmet. The black and blue dotted horizontal lines represent the mean value of all helmets and conventional helmets, respectively.

Figure 5 presents the kinematic-based injury metrics, PTA, PRA, PRV and BrIC, for all impacts and helmets. The results from the three repeats across all metrics have small differences for all helmets and impact locations. The mean values of the injury metrics from the repeats are shown in Tables A1–A4 in the supplementary material Appendix 1. For each helmet at each impact location, we computed the coefficient of variation (CV) of the three repeats. Most CVs were small and only in a few test conditions, CV was above 10% (Tables A1–A4). This was due to the inconsistent performance of some helmets at certain impact locations. For example, in terms of PTA, only B.Q-MIPS and I.A-MIPS at impact location 5 (rear-side impact) had CVs above 10%, which suggested that the performance of MIPS helmets was not very consistent at this impact location.

### The Effects of Impact Location on Kinematics-Based Injury Metrics

The two-way ANOVA showed that both the helmet type and impact location have significant effects on PTA, PRA, PRV and BrIC (*p* < 0.001). Hence, we first studied the effects of impact location on each injury metric. In Figure 5, we show the mean value of the injury metric for each impact location using a broken black line to help with the comparisons. For PTA, impact 2 and 5 (rear impacts) produced lower values than other impact locations. The post-hoc test confirmed that impact 2 and 5 are significantly different from the other three impacts (*p* < 0.001), and there is no significant difference between impacts 1, 3 and 4 (*p* > 0.9).

For PRA, impact 5 and 2 (rear impacts) produced higher values than the other three impacts. The post-hoc test showed that Impact 2 and 5 are significantly different from other impact locations (*p* < 0.01), and there is no significant difference between PRA in impacts 1, 3 and 4 (*p* > 0.9).

For PRV, impact 4 (frontal impact) generated the highest values and impact 3 (side impact) produced the lowest values. The post-hoc test showed that all impacts are significantly different (*p* < 0.001), except impact 1 and 2 (*p* = 0.325).

BrIC followed a trend like PRV. Impact 4 produced the highest values and impact 3 produced the lowest values. Impact 1 and 2 are not significantly different (*p* = 0.797), but all the other impact location comparisons are significantly different (*p* < 0.001).

### The Effects of Technology on Kinematics-Based Injury Metrics

Next, we investigated how the three technologies (MIPS, Flex and ODS) affect the kinematics-based injury metrics. The one-way ANOVA with technology as the factor shows that the helmet technology has a significant effect on all four injury criteria (*p* < 0.05).

For PTA, the post-hoc test shows that only helmets with MIPS have significantly lower PTA values than conventional helmets (on average 8.5% lower across all impact locations, p=0.036). Helmets with Flex and ODS technologies do not have statistically different PTA compared with conventional helmets (*p* > 0.3). In addition, their effect on PTA is not consistent in different impact locations. For example, in impact locations 1, 2 and 5, Flex produces lower mean PTA than conventional helmets while in impacts 3 and 4, it produces higher mean PTA.

For PRA, the post-hoc test shows that MIPS and Flex significantly reduce PRA compared with conventional helmets (MIPS: on average 31.2% lower across all impact locations, *p* < 0.001, Flex: on average 19.3% lower across all impact locations, *p* < 0.001). In addition, MIPS reduces the PRA compared to Flex (on average 14.8% lower across all impact locations, *p* = 0.005). The ODS technology slightly increases the mean PRA than conventional helmets, but the difference is not significant (on average 0.36% higher across all impact locations, *p* = 1.000). ODS has lower mean PRA than conventional helmets in impact 1 and 4, but it has higher PRA in the other three impact locations.

For PRV, the post-hoc test shows that only MIPS helmets have significantly lower value than conventional helmets (on average 11.5% lower across all impact locations, *p* < 0.001). Although the Flex and ODS produce lower PRV than conventional helmets, their effect is not significant (*p* > 0.6) and it is inconsistent across impact locations.

Finally, for BrIC, the post-hoc test shows that only MIPS helmets have significantly lower value than conventional helmets (on average 10.7% lower across all impact locations, *p* = 0.011). Flex and ODS do not have different results compared with conventional helmets (*p *> 0.8).

### Predicted Strain Across the Brain in Oblique Impacts

**Figure 6:**
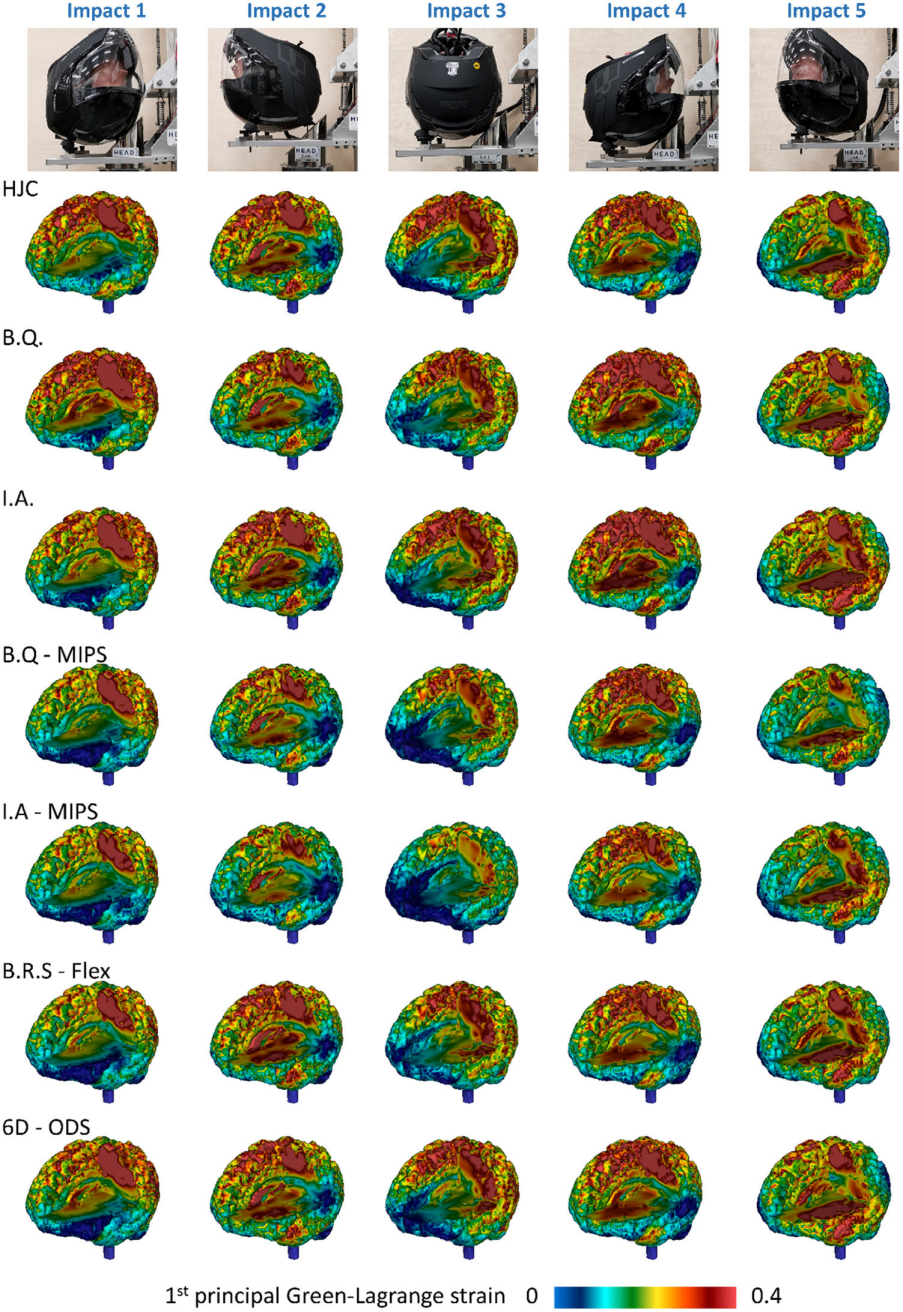
Strain distribution across the brain predicted by the human head FE model. The results for tests with the largest 90th percentile overall strain are shown here. Part of the brain is masked to show the strain distribution inside the brain.

We predicted the distribution of maximum first principal Green-Lagrange strain across the brain for all impacts. Figure 6 shows the contours for the tests that led to the largest 90th percentile strain among the three repeats. As can be seen, a large volume of the brain undergoes strains more than 0.2 in all impact locations. Impact 1 and 4 (frontal impacts) produce large strains in the parietal lobe and corpus callosum (CC). Impacts 2 and 5 (rear impacts) affect more areas including the temporal lobe. Impact 3 (side impact) produces large strains in cortical and subcortical regions, but its effects are not as widespread as the other impacts. It particularly produces lower strains in the CC and frontal lobe.

Figure 6 also shows that helmets have large effects on the strain distribution. An interesting comparison can be made between B.Q. and I.A. and their pairs that are equipped with MIPS. MIPS reduces the strain across the brain with a clearer effect for I.A. helmet than B.Q.

**Figure 7:**
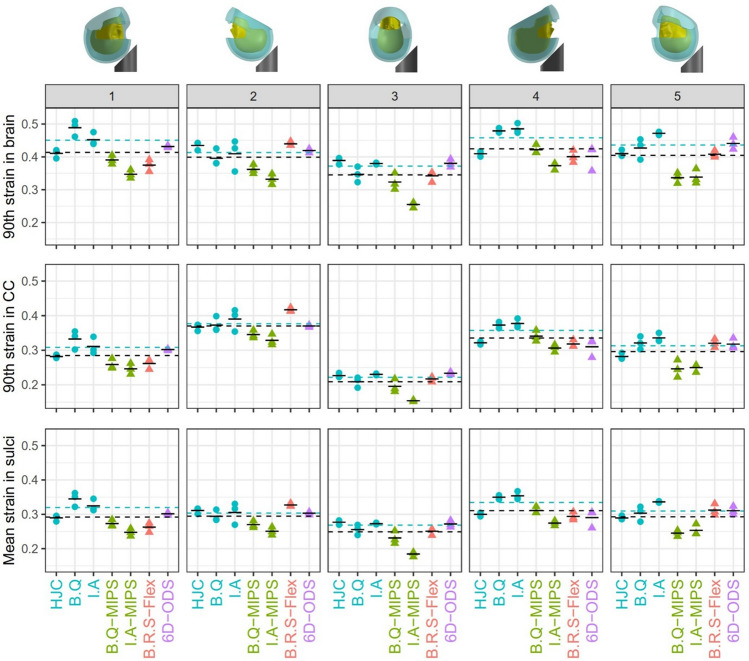
Strains for all helmets at all impact locations. For the entire brain and corpus callosum, the 90th percentile strain were calculated. For the sulci region, the mean strain values were calculated. The plot includes three repeats (markers) and the mean value (broken black line) of each helmet. The black and blue dotted horizontal lines represent the mean strain value of all helmets and that of conventional helmets, respectively.

To quantify these comparisons, we calculated the 90th percentile strain across the entire brain and CC and the mean strain in sulci (Figure 7). The mean values of the brain strains from three repeats are shown in Tables A5–A7 in Appendix 1. For each helmet at each impact location, we computed the CV of the three repeats’ simulations. All CVs were small and less than 10% (Tables A5–A7), suggesting good repeatability among simulation results.

### The Effects of Impact Location on Brain Strain

The two-way ANOVA results showed that both helmet type and impact location have significant effects on the overall strain and strain in CC and sulci (*p* < 0.001). We first investigated the effects of impact location on brain strain. Impact 3 (side impact) produced lowest strain across the whole brain, in CC and in sulci compared with the other impact locations (*p* < 0.001). Impact 2 (rear impact) produced the highest strain in CC (*p* < 0.001). The other comparisons did not show any significant differences.

### The Effects of Helmet Technology on Brain Strain

Next, we investigate how the three technologies affect the brain strains. The one-way ANOVA using the technology as the factor shows that the helmet technology has a significant effect on the three strain measures (*p* < 0.01). For strain across the entire brain, the post-hoc test shows that MIPS has significantly lower values than conventional helmets (on average 18.3% lower across all impact locations, *p* < 0.001). Flex also reduces strain significantly compared with conventional helmets (on average 7.7% lower across all impact locations, *p *= 0.046), but ODS does not (*p *= 0.799). MIPS consistently reduces the mean values in all impact locations. Flex reduces strain in all impacts except impact 2.

For the CC strain, the post-hoc test shows that only MIPS produces significantly lower values than the conventional helmets (on average 15.4% lower across all impact locations, *p *= 0.005). In addition, its effect is consistent across all impact locations. This contrasts with Flex and ODS, which have inconsistent effects on strain in CC and overall do not significantly reduce CC strain (*p* > 0.9).

For the mean strain in sulci, the post-hoc test also shows that only MIPS significantly reduces strain than the conventional helmets (on average 17.3% lower across all impact locations, *p* < 0.001) and its effect is consistent across all impact locations. Flex and ODS do not have consistent effects on strain in sulci and overall do not produce different results compared with conventional helmets (*p* > 0.2).

## Discussion

We determined the performance of 7 modern motorcycle helmets under oblique impacts at different locations, including conventional helmets and helmets fitted with rotation management technologies MIPS, Flex and ODS. Our results show a wide range of head and brain responses, measured with PTA, PRA, PRV, BrIC and brain strain, depending on the helmet type and impact location. The helmets fitted with MIPS technology were effective in reducing injury metrics based on head rotation (PRA, PRV and BrIC) and brain strain. The Flex technology was effective in reducing PRA and overall brain strain but not in relation to other injury metrics. The ODS however was not effective considering different injury metrics.

Our results showed that impact location has significant effects on injury metrics, but its effect is not consistent across the metrics. For example, impact 2 (rear impact) produced the lowest PTA but the highest PRA than other locations. Impact 3 (side impact) and impact 4 (frontal impact) produced similar PTA and PRA values. However, the PRV and BrIC in impact 3 were much lower than impact 4. These differences can be due to the difference in the helmet geometry and liner thickness at different impact locations, affecting the contact force vector and head kinematics.^47^ Another potential reason for the differences is the constraint between the helmet and headform. Previous study suggested that constraints between the helmet and headform in different anatomical planes allow different level of headform-helmet relative rotation about different axis.^1^ The impact location also showed significant effects on brain strain. This can be due to different head kinematics and brain responses to rotations about different axes. It has been shown that head rotations about the inferior–superior (*z*) axis produce larger brain strain than rotation about the other axes.^54,56^ This may explain why impact 1 (front-side impact) and impact 5 (rear-side impact) produced higher overall brain strain than impact 3 (side impact). It is also noteworthy that impact 2 (rear) and impact 4 (front) produced largest strains in CC than other impact locations. These results show that different impact locations have different effects on injury metrics. Hence, we recommend the inclusion of different impact locations and injury metrics in future helmet standards to better evaluate the helmet performance.

We used four kinematics-based injury metrics to evaluate the performance of helmets in reducing the risk of different pathologies seen in motorcycle accidents. PTA is used in all helmet standards as the head injury criterion. Studies on post-mortem human subjects (PMHS) and animals have shown that PTA can predict the risk of skull fractures and focal brain injuries.^3,25^ The other metrics, PRA, PRV and BrIC, are based on the rotational motion of the head. PMHS and animal experiments have shown that PRA can predict the risk of SDH.^14,22^ PRV and BrIC are shown to predict the risk of DAI based on experiments on animals and finite element prediction of brain strain.^40,54^ Interestingly the ECE22.06 standard now requires recording the PRA and BrIC and proposes a limit for them. Here we have not compared our results with these limits because we used a HIII headform, whose physical properties, particularly the coefficient of friction, is different to the EN960 headform used in ECE22.06. Neither have we compared our results with the threshold values suggested for different injury metrics in previous studies. Hence, our study provides a comparison between the performance of helmets in reducing the risk of different types of injuries rather than the absolute risk of injuries associated with each helmet.

Our oblique impact test method is identical to that in ECE22.06 standard, except that we used a HIII 50th percentile male dummy headform rather than the EN960 headform specified in ECE22.06. This choice was made due to the better fidelity of the HIII headform in terms of mass and mass moments of inertia. Connor *et al*.^12^ used CT scans of 56 subjects to determine the physical properties of the living human head, including the head circumference, mass and moments of inertia (MoIs), and to determine relationships describing mass and MoIs as functions of head circumference. We used these functions to test whether the mass and MoIs of the EN960 and HIII headforms are close to those of the population used in Ref. 12. We found that the HIII headform’s properties are closer to the human head than EN960 (size J). For instance, we found that the HIII headform is 6.3% heavier but the EN960-J headform is 10%. For MoI about the *X* axis (*I*_xx_), the EN960-J headform had a value 27% higher and the HIII headform 24% lower. In terms of MoI about the *Y* axis (*I*_yy_), EN960-J had a value 44% larger while the HIII headform’s value was only 8.6% larger. For MoIs about the *Z* axis (*I*_zz_), both headforms had much larger values (EN960-J: 35% higher and HIII: 54% higher). In our experiments under the 5 impact locations, the dominant rotational accelerations are about the *X* and *Y* axes, where the *I*_xx_ and *I*_yy_ play a key role. Future work should focus on the development of headforms with more biofidelic mass and MoIs, particularly if new impact locations will be introduced to produce dominant rotation about the *Z* axis.

We used the Imperial College human head FE model, which includes fine details of brain anatomy, to predict strain distribution across the brain. Mechanical strain is shown to predict brain tissue pathologies, such as axonal injuries, vascular injuries, neuronal cell death and inflammation.^5,16,19,24,36,48^ Here, we not only used a global measure of strain, but also determined strain in two regions of interest, corpus callosum and sulci, like in a previous work on bicycle helmets.^1^ Corpus callosum is the largest white matter tract and often damaged after TBI, and its damage is associated with cognitive impairment.^34,37^ Sulci is the location of CTE pathology, and post-mortem and MRI studies have shown abnormalities in this region in long-term survivors of repetitive and single head impacts.^4,24,33,41^ The inclusion of sulci in the model allowed us to determine strain in this region for different impact locations and helmet technologies. The advantage of using the modelling approach is that it provides novel information on the effect of helmets on different regions of the brain, which should be a direction for improving helmet design in future.

We selected 7 helmets, 5 of which have been tested under the SHARP rating program. The HJC and B.R.S-Flex helmets were rated as 5 stars and the B.Q, I.A and B.Q-MIPS were rated as 3 stars. However, these ratings did not correlate with our test results. For example, our results showed B.Q-MIPS (3 star) had a better performance across all the injury metrics and brain strains than HJC (5 star). This difference is primarily due to the difference in helmet testing methods. We conducted oblique impacts using a 45° anvil, which is a common scenario in motorcycle collisions.^10^ The difference between 1-star and 5-star SHARP rating in terms of reducing risk of fatal injuries is nearly 60%. In SHARP, the rating is based on 30 linear impacts and two oblique impacts on a 15° anvil. The oblique impact tests results are used to calculate the coefficient of friction at the helmet shell-road interface, which is used to scale the PTA measured in linear impacts.^43^ However, rotational accelerations are not measured in the oblique impact tests. The SHARP rating scheme is currently undergoing a review to possibly incorporate the new advances in the field of TBI biomechanics and helmet testing.

Another interesting observation was that there was no association between helmet performance in oblique impacts and helmet price. The helmet price was mainly determined by the technology and the shell material, with CFRP shell helmets being more expensive than helmets with polycarbonate shells. For example, the price of 6D-ODS helmet was 2–3 folds of the MIPS helmets. However, the 6D-ODS helmet had inferior performance than the B.Q-MIPS and I.A-MIPS helmets across all injury metrics. Safety is a key factor in selecting a suitable helmet. Hence, end-users will substantially benefit from reports on head protection performance of helmets in realistic impact conditions.

This study has some limitations. We only included three conventional helmets in our baseline group, which may not reflect the average performance of conventional helmets. This was primarily due to the high cost of motorcycle helmets (for each helmet, we required six samples). In our future work, we will test more helmets to expand the baseline group. We only tested three rotation management technologies incorporated in motorcycle helmets available in the UK market. These technologies follow a similar strategy, i.e., facilitating relative motion between the helmet and head to reduce head rotation. Other strategies, such as inflatable helmets and cellular liners, are currently only available in bicycle helmets.^1^ In addition, only the two helmets with MIPS technology have their corresponding versions without MIPS. All the other helmets have different geometries, which may affect the headform kinematics during impact. For instance, the initial impact point on the helmet shell may be different among helmets with different shell shapes, and such a difference may have an effect on the headform kinematics. Another limitation is that the coefficient of friction between the HIII headform and helmet liner is higher than that between the human scalp and helmet liner.^55^ High coefficient of friction may lead to higher PRA, PRV and BrIC values. Similar to several previous studies,^1,7,8^ we used the HIII headform due to its mass and moments of inertia being closer to that of the average human head.^30^ The EN960 headform, which is used by ECE22.06, has a lower coefficient of friction than the HIII headform, but it has higher mass and moments of inertia than the average human head.^12^ Using stocking to cover the HIII headform is a potential way to reduce surface friction.^8,9,31^ However, the CoF between stocking covered headforms and helmet liner has not been reported previously. In addition, there may be differences in the material, thickness and covering method of stockings in different studies affecting the results. Therefore, using a stocking-covered HIII headform without full characterisation of the friction properties will introduce new uncertainties, affecting the reproducibility of the study. Future work should focus on developing headforms with better coefficient of friction, which is robust and fully characterised. Finally, the five impact locations are within the transverse plane, which produced headform rotation mainly about X and Y axis. These impact locations cannot generate headform rotation about the Z axis, which is shown to produce larger strains in the brain.^20^ In our future work, we will extend the oblique impact tests to study the helmet protective performance for headform rotation about Z axis.

## Conclusions

In summary, we compared the head and brain protection performance of several modern motorcycle helmets under oblique impacts against a 45° anvil at 8m/s and five locations. The results showed that the impact location has a significant effect on the helmet protective performance. However, this effect is not consistent considering different kinematics-based injury metrics and strain distribution across the brain, corpus callosum and sulci. Among the three rotational management technologies, MIPS was effective in reducing all injury metrics and brain strain compared with conventional helmets. Flex was effective in reducing PRA only and ODS was not effective in reducing any injury metrics. In addition, there was no association between price and performance of the studied helmets, with very expensive helmets not providing better protection than low price helmets. This study shows the importance of using different impact locations and injury metrics when assessing head protection effects of helmets. It also provides new data on the performance of modern motorcycle helmets. These results can help with improving helmet design and developing standard and rating test methods.

## Supplementary Information

Below is the link to the electronic supplementary material.

Supplementary file1 (PDF 655 kb)
