# Supplementary material for: The Protective Performance of Modern Motorcycle Helmets Under Oblique Impacts
Source: Ann Biomed Eng. 2022 Apr 13;50(11):1674–88. doi: 10.1007/s10439-022-02963-8 (PMC9652253; doi:10.1007/s10439-022-02963-8)
Supplement: Supplementary file 1 — Supplementary file1 (PDF 655 kb) [file 10439_2022_2963_MOESM1_ESM.pdf]

Appendix 1

Table A1 The PTA (g) (mean value and CV of the three repeats) of all helmets at different impact points.

Helmets	Technology	Impact locations					Mean ± SD
		1	2	3	4	5	
HJC	None	166.5 (2.7%)	134.7 (4.9%)	163.2 (2.9%)	157.1 (0.7%)	171.7 (0.7%)	158.6±12.9
B.Q		165.6 (3.6%)	136.1 (3.6%)	148.8 (4.8%)	161.3 (1.4%)	170.6 (2.3%)	156.5±12.5
I.A		159.5 (3.9%)	131.3 (6.0%)	159.1 (2.1%)	159.5 (0.5%)	119.3 (7.2%)	145.7±17.1
B.Q-MIPS	MIPS	154.5 (2.2%)	139.8 (3.3%)	150.7 (6.8%)	151.3 (1.9%)	155.3 (11.8%)	150.3±5.6
I.A-MIPS	MIPS	150.7 (5.2%)	118.6 (5.7%)	135.4 (4.2%)	147.1 (1.7%)	102.3 (10.6%)	130.8±18.1
B.R.S-Flex	Flex	155.8 (3.4%)	99.5 (5.7%)	174.1 (1.8%)	163.9 (6.9%)	125.4 (8.2%)	143.7±27.4
6D-ODS	ODS	156.7 (3.9%)	135.7 (3.0%)	173.6 (8.1%)	165.4 (3.9%)	120.5 (3.5%)	150.4±19.6
Mean ± SD		158.5± 5.4	127.9± 13.2	157.8± 13.0	157.9± 6.2	137.9± 25.6	

Table A2 The PRA (rad/s^2) (mean value and CV of the three repeats) of all helmets at different impact point.

Helmets	Technology	Impact locations					Mean ± SD
		1	2	3	4	5	
HJC	None	8700 (12.8%)	11020 (1.6%)	10328 (2.1%)	8739 (9.1%)	10263 (4.3%)	9810±929
B.Q		11268 (5.0%)	9202 (3.7%)	8095 (11.8%)	10119 (5.2%)	11198 (4.3%)	9976±1210
I.A		9498 (6.7%)	10339 (9.9%)	10393 (6.3%)	10067 (0.6%)	11186 (1.2%)	10297±546
B.Q-MIPS	MIPS	7578 (7.3%)	7502 (4.6%)	7202 (5.3%)	8013 (2.0%)	8059 (2.1%)	7671±324
I.A-MIPS	MIPS	5868 (7.4%)	6929 (4.4%)	4717 (5.6%)	7052 (6.3%)	6028 (2.8%)	6119±844
B.R.S-Flex	Flex	7236 (0.7%)	9086 (2.5%)	8574 (4.0%)	7352 (6.8%)	8213 (3.6%)	8092±709
6D-ODS	ODS	9125 (5.4%)	10985 (4.0%)	10702 (4.3%)	8483 (12.9%)	10664 (6.8%)	9992±997
Mean ± SD		8468± 1624	9295± 1502	8573± 1996	8546± 1119	9373± 1826	

Table A3 The PRV (rad/s) (mean value and CV of the three repeats) of all helmets at different impact points.

Helmets	Technology	Impact locations					Mean ± SD
		1	2	3	4	5	
HJC	None	39.1 (1.9%)	39.9 (2.6%)	35.1 (1.5%)	42.4 (1.4%)	36.1 (2.0%)	38.5±2.6
B.Q		45.5 (4.6%)	39.5 (2.8%)	33.3 (4.2%)	47.8 (1.2%)	38.1 (4.1%)	40.8±5.2
I.A		42.5 (3.4%)	42.0 (5.0%)	34.9 (1.0%)	48.5 (3.4%)	42.4 (1.3%)	42.1±4.3
B.Q-MIPS	MIPS	38.1 (1.5%)	37.3 (1.1%)	30.6 (4.9%)	44.5 (4.4%)	30.2 (6.8%)	36.1±5.3
I.A-MIPS	MIPS	38.3 (2.7%)	36.5 (3.2%)	26.7 (2.1%)	41.7 (1.9%)	34.4 (4.7%)	35.5±5.0
B.R.S-Flex	Flex	38.9 (0.5%)	44.0 (1.1%)	34.2 (1.3%)	42.5 (2.9%)	37.2 (0.8%)	39.4±3.6
6D-ODS	ODS	41.7 (1.0%)	39.1 (0.9%)	34.8 (2.5%)	39.9 (5.2%)	39.3 (3.1%)	39.0±2.3
Mean ± SD		40.6± 2.6	39.8± 2.4	32.8± 2.9	43.9± 3.0	36.8± 3.6	

Table A4 The BrIC (mean value and CV of the three repeats) of all helmets at different impact points.

Helmets	Technology	Impact locations					Mean ± SD
		1	2	3	4	5	
HJC	None	0.69 (0.9%)	0.71 (2.3%)	0.55 (1.9%)	0.76 (1.4%)	0.62 (1.7%)	0.67±0.07
B.Q		0.80 (5.1%)	0.70 (2.7%)	0.53 (2.6%)	0.85 (1.5%)	0.68 (4.1%)	0.71±0.11

I.A		0.76 (4.9%)	0.75 (4.9%)	0.54 (1.6%)	0.87 (3.5%)	0.72 (0.8%)	0.73±0.11
B.Q-MIPS	MIPS	0.66 (2.0%)	0.66 (1.1%)	0.47 (5.3%)	0.80 (4.2%)	0.57 (3.6%)	0.63±0.11
I.A-MIPS	MIPS	0.68 (3.4%)	0.65 (3.2%)	0.42 (2.6%)	0.74 (2.1%)	0.61 (3.2%)	0.62±0.11
B.R.S-Flex	Flex	0.68 (0.5%)	0.78 (1.0%)	0.53 (1.6%)	0.76 (2.9%)	0.67 (1.8%)	0.68±0.09
6D-ODS	ODS	0.73 (0.7%)	0.69 (0.8%)	0.55 (3.5%)	0.71 (4.8%)	0.68 (2.6%)	0.67±0.06
Mean ± SD		0.71±<br>0.05	0.71±<br>0.04	0.51±<br>0.05	0.78±<br>0.05	0.65±<br>0.05	

Table A5 The 90th strain of the entire brain (mean value and CV of the three repeats) of all helmets at different impact points.

**Helmets**	**Technology**	**Impact locations**					**Mean ± SD**
		**1**	**2**	**3**	**4**	**5**	
HJC		0.41 (2.6%)	0.44 (2.4%)	0.39 (2.2%)	0.41 (1.6%)	0.41 (2.0%)	0.41±0.02
B.Q	None	0.49 (4.1%)	0.40 (5.3%)	0.35 (5.6%)	0.48 (1.3%)	0.43 (6.0%)	0.43±0.05
I.A		0.45 (3.6%)	0.41 (9.5%)	0.38 (0.6%)	0.49 (2.6%)	0.47 (0.8%)	0.44±0.04
B.Q-MIPS	MIPS	0.39 (2.8%)	0.36 (3.2%)	0.32 (6.4%)	0.42 (2.7%)	0.34 (3.9%)	0.37±0.04
I.A-MIPS	MIPS	0.35 (3.0%)	0.33 (4.0%)	0.26 (2.9%)	0.37 (2.5%)	0.34 (5.4%)	0.33±0.04
B.R.S-Flex	Flex	0.38 (4.0%)	0.44 (1.3%)	0.34 (4.1%)	0.40 (3.8%)	0.41 (2.1%)	0.39±0.03
6D-ODS	ODS	0.43 (0.6%)	0.42 (1.2%)	0.38 (2.6%)	0.40 (7.7%)	0.44 (3.4%)	0.41±0.02
Mean ± SD		0.41±<br>0.04	0.40±<br>0.04	0.35±<br>0.04	0.42±<br>0.04	0.41±<br>0.05	

Table A6 The 90th strain of the corpus callosum (mean value and CV of the three repeats) of all helmets at different impact points.

**Helmets**	**Technology**	**Impact locations**					**Mean ± SD**
		**1**	**2**	**3**	**4**	**5**	
HJC		0.28 (1.4%)	0.37 (2.2%)	0.23 (2.4%)	0.32 (1.1%)	0.28 (2.6%)	0.30±0.05
B.Q	None	0.33 (6.7%)	0.37 (4.9%)	0.21 (5.9%)	0.37 (2.0%)	0.32 (4.8%)	0.32±0.06
I.A		0.31 (6.5%)	0.39 (6.7%)	0.23 (0.8%)	0.38 (2.8%)	0.34 (3.1%)	0.33±0.06
B.Q-MIPS	MIPS	0.26 (4.8%)	0.35 (2.7%)	0.20 (8.1%)	0.34 (3.8%)	0.25 (8.3%)	0.28±0.06
I.A-MIPS	MIPS	0.25 (5.2%)	0.33 (4.0%)	0.15 (3.7%)	0.31 (2.8%)	0.25 (3.8%)	0.26±0.06
B.R.S-Flex	Flex	0.26 (4.6%)	0.42 (1.1%)	0.22 (2.9%)	0.32 (2.7%)	0.32 (3.1%)	0.31±0.07
6D-ODS	ODS	0.30 (6.6%)	0.37 (7.3%)	0.23 (1.4%)	0.31 (7.1%)	0.32 (3.9%)	0.31±0.04
Mean ± SD		0.28±<br>0.03	0.37±<br>0.03	0.21±<br>0.03	0.34±<br>0.03	0.30±<br>0.03	

Table A7 The mean strain of the sulci (mean value and CV of the three repeats) of all helmets at different impact points.

**Helmets**	**Technology**	**Impact locations**					**Mean ± SD**
		**1**	**2**	**3**	**4**	**5**	
HJC		0.29 (2.6%)	0.31 (2.3%)	0.28 (1.9%)	0.30 (1.5%)	0.29 (1.7%)	0.29±0.01
B.Q	None	0.35 (4.9%)	0.29 (4.7%)	0.26 (4.8%)	0.35 (1.4%)	0.30 (6.0%)	0.31±0.03
I.A		0.33 (4.6%)	0.31 (8.5%)	0.27 (0.7%)	0.35 (2.7%)	0.34 (0.5%)	0.32±0.03
B.Q-MIPS	MIPS	0.27 (3.0%)	0.27 (3.0%)	0.23 (6.6%)	0.31 (2.7%)	0.25 (2.9%)	0.27±0.03
I.A-MIPS	MIPS	0.25 (3.6%)	0.25 (3.8%)	0.18 (3.1%)	0.27 (2.1%)	0.25 (5.3%)	0.24±0.03
B.R.S-Flex	Flex	0.26 (4.2%)	0.33 (1.2%)	0.25 (3.3%)	0.29 (3.4%)	0.31 (4.4%)	0.29±0.03
6D-ODS	ODS	0.30 (1.1%)	0.30 (1.2%)	0.27 (2.9%)	0.29 (7.3%)	0.31 (3.2%)	0.30±0.01
Mean ± SD		0.29±<br>0.03	0.29±<br>0.02	0.25±<br>0.03	0.31±<br>0.03	0.29±<br>0.03	

Appendix 2

Verification of using the masking tape to fix the helmeted headform

In the tests, we used masking tape to temporarily fix the helmet onto the platform. This maintained the helmet's position and orientation during the free fall. The width of the masking tape was 48mm and half-width of the tape was pre-cut at several locations to ensure an easy tear during the impact. To determine the effect of the masking tape on the headform kinematics during the oblique impact, we used bicycle helmets, rather than motorcycle helmets, because the headform is more visible when fitted with bicycle helmets. This allowed us to compare headform movements across different test conditions.

Table A8 shows the test matrix. We used 6 helmet samples and conducted 18 oblique tests at 6m/s impact velocity. In 9 tests, the helmets were fixed with the masking tape and in the other 9 tests, no tapes were used. With each helmet sample, we conducted three impacts at the front, side and rear-side of the helmet. For the rear-side impact, the angle between the headform's sagittal plane and the anvil middle plane was 45° in order to produce a more complex impact response than a pure rear impact. The tests were repeated three times using a new helmet.

We first compared the head movement between the with/without tape experiments using the video recorded by the high-speed video camera. As shown in Figure A1, we selected the snapshots at the same time, which is 29.4ms after camera triggering. At this time, the impact between the helmet and the anvil has finished. We also overlaid the snapshots of the tests with the masking tape (50% transparency) on the those without. The comparisons show that the headform and helmet motion are very similar between the tests with and without the masking tape.

Next, we processed the acceleration data of each test to obtain the four brain injury metrics (PTA, PRA, PRV and BrIC). We conducted one-way ANOVA with the presence of the masking tape as the factor to determine the effects of the tape on the injury metrics. The results show that for all the three impact locations, using the masking tape did not have a significant effect on the injury metrics (Table A9-A12).

In summary, these results verified that using the masking tape did not affect the headform kinematics. Compared with these tests, motorcycle helmets were tested at a higher speed (8m/s), leading to much higher impact force and energy. As the tearing force of the masking tape is the same, the effects of the on the head kinematics in motorcycle helmet tests should have been negligible.

Table A8 The test matrix for investigating the effects of using masking tape.

**Test number**	**Helmet sample**	**Impact location**	**Masking tape**
1	1	Front	Yes
2	2	Front	Yes
3	3	Front	Yes
4	4	Front	No
5	5	Front	No
6	6	Front	No
7	1	Side	Yes
8	2	Side	Yes

9	3	Side	Yes
10	4	Side	No
11	5	Side	No
12	6	Side	No
13	1	Rear-side	Yes
14	2	Rear-side	Yes
15	3	Rear-side	Yes
16	4	Rear-side	No
17	5	Rear-side	No
18	6	Rear-side	No

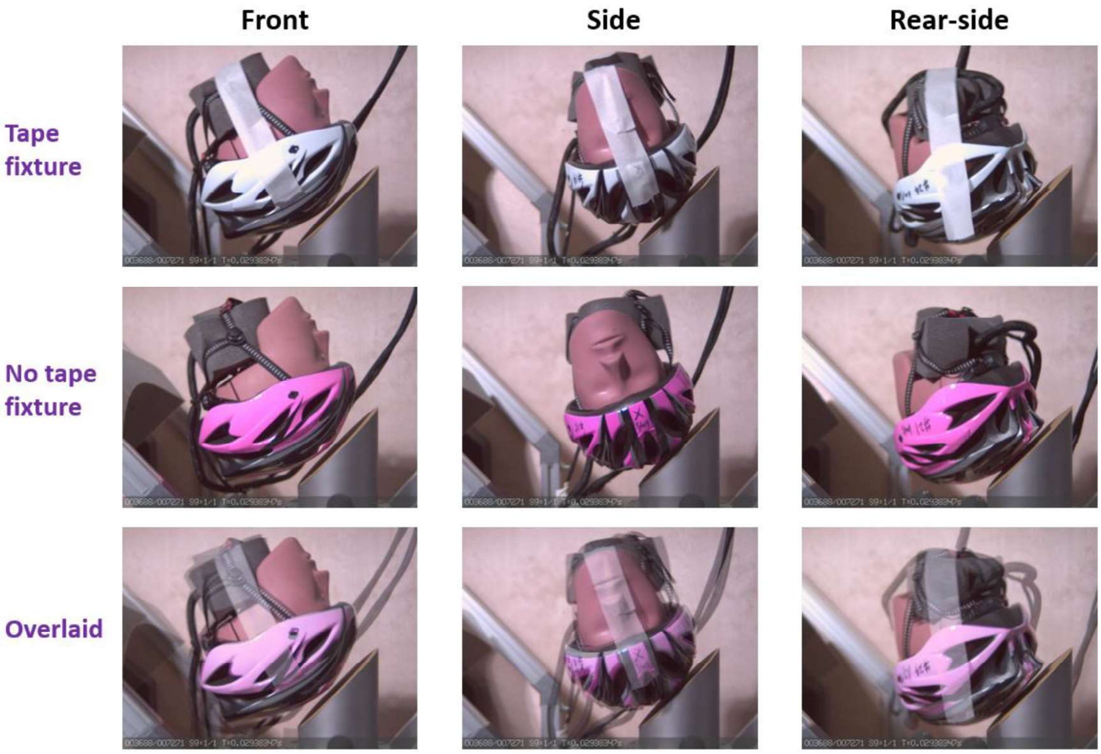

Figure A1 Snapshots (29.4ms after camera triggering) from the high-speed videos of the tests with and without masking tape, and the snapshots of the tests with masking tape (50% transparency) overlaid on those without masking tape fixture.

Table A9 The PTA (g) of all tests at different impact locations.

Impact location	Front		Side		Rear-side	
Tape	Yes	No	Yes	No	Yes	No
Repeat 1	129.5	123.0	124.6	121.6	68.0	83.5
Repeat 2	121.3	128.1	124.0	124.4	68.7	73.4
Repeat 3	125.4	125.5	122.9	120.5	79.8	61.4
One-way ANOVA	p=0.961		p=0.263		p=0.939	

Table A10 The PRA (rad/s^2) of all tests at different impact locations.

Impact location	Front		Side		Rear-side	
Tape	Yes	No	Yes	No	Yes	No
Repeat 1	8607	8290	5345	8305	6268	7796
Repeat 2	8280	9094	5886	6736	6648	7670
Repeat 3	8790	8466	5571	5715	7699	6964
One-way ANOVA	p=0.85		p=0.162		p=0.293	

Table A11 The PRV (rad/s) of all tests at different impact locations.

Impact location	Front		Side		Rear-side	
Tape	Yes	No	Yes	No	Yes	No
Repeat 1	37.6	37.6	26.1	33.3	36.5	39.6
Repeat 2	37.2	38.1	27.2	29.5	38.7	41.5
Repeat 3	37.2	37.2	26.6	27.5	40.0	41.6
One-way ANOVA	p=0.355		p=0.115		p=0.109	

Table A12 The BrIC of all tests at different impact locations.

Impact location	Front		Side		Rear-side	
Tape	Yes	No	Yes	No	Yes	No
Repeat 1	0.67	0.67	0.42	0.54	0.65	0.71
Repeat 2	0.66	0.68	0.47	0.48	0.69	0.74
Repeat 3	0.66	0.67	0.42	0.44	0.71	0.77
One-way ANOVA	p=0.239		p=0.229		p=0.0779	
